# Complete genome sequence of *Escherichia marmotae* F12YCO47 isolated from a healthy human fecal sample

**DOI:** 10.1128/mra.01241-24

**Published:** 2025-03-25

**Authors:** Daniel Ho, Sucharita Gali, Hui Hui Phua, Natalia Seabra, Wayne S. Cutfield, Justin M. O'Sullivan, the Gut Bugs Study Group, Brooke C. Wilson, Amila S. N. W. Pahalagedara

**Affiliations:** 1 Liggins Institute, University of Auckland 56386, Auckland, New Zealand; 2 Maurice Wilkins Centre for Molecular Biodiscovery 428622 , Auckland, New Zealand; 3 MRC Lifecourse Epidemiology Unit, University of Southampton 47980, Southampton, United Kingdom; 4 Singapore Institute for Clinical Sciences, Agency for Science, Technology and Research (A*STAR) 303181, Singapore, Singapore; University of Maryland School of Medicine, Baltimore, Maryland, USA

**Keywords:** *Escherichia marmotae*, human gut microbiome, genome sequencing, fecal microbiota

## Abstract

*Escherichia marmotae* has been found in animals and the environment. Here, we isolated it from a healthy human fecal sample. The 4.91 Mb circular genome (GC content = 50.34%) is associated with three plasmids: pF12YCO47-2 (89.9 kb, 50.2% GC), pF12YCO47-3 (47.9 kb, 44.3% GC), and pF12YCO47-1 (95.4 kb, 47.2% GC).

## ANNOUNCEMENT

*Escherichia marmotae*, a gram-negative bacterium in *Enterobacteriaceae*, was first isolated from Himalayan marmot (*Marmota himalayana*) feces (1). Initially classified as *Escherichia* cryptic clade V, it was considered a naturalized member of the genus with limited human presence (2). Since its new classification, *E. marmotae* has been found in wild animals and environmental sources (3, 4) and identified as a likely pathogen of invasive human infections (2).

F12YCO47 was isolated from the fecal sample of a healthy 26-yearold woman during an obesity FMT study (ethics: 16/NTA/172) (5). F12YCO47 was originally obtained by culturing a serially diluted fecal sample (1:10) aerobically on yeast casitone fatty acids agar (YCFA, GMExpression, Australia) at 37°C for 24 hours. The F12YCO47 colony was picked and sub-cultured on YCFA plates (37°C, overnight) several times to obtain a pure colony. MALDI-TOF MS (Bruker Daltonic, Germany) confirmed a high confident match (score 2.61) to *E. marmotae*.

For genome sequencing, F12YCO47 was revived (37°C, overnight) on sheep blood agar (Fort Richard, New Zealand). One colony was picked and grown in tryptic soy broth (37°C, overnight). DNA was extracted (1 mL overnight culture; QIAamp PowerFecal Pro DNA Kit [Qiagen, Germany]; according to the manufacturer’s recommendations), and a DNA library was prepared (Oxford Nanopore Technologies [ONT] ligation sequencing kit V14 [SQL-LSK114], according to the manufacturer’s instructions). Sequencing was performed using an R10.4 flow cell on an ONT PromethION 2 Solo device. Base calling was performed using Dorado version 7.3.9 (https://github.com/nanoporetech/dorado) with the super accurate model in MinKNOW version 24.02.10 (ONT, UK). The Epi2me-labs/wf-bacterial-genomes pipeline (https://github.com/epi2me-labs/wf-bacterial-genomes) was used for genome assembly (*Q*-score cutoff: 15). The genome was assembled (Flye version 2.9.5-b180 [6]), polished (Medaka [https://github.com/nanoporetech/medaka]), and annotated (NCBI Prokaryotic Genome Annotation Pipeline [PGAP] version 6.8 [7]). Flye version 2.9.5-b180 identified all contigs as circular based on loop formation in the repeat graph of the assembly. Default parameters were used for all software unless otherwise specified.

Genome quality was assessed (CheckM version 1.2.3 [8] and QUAST version 5.2.0 [9]). CheckM showed 100% completeness and minimal contamination (0.08%) (8). QUAST confirmed the assembly quality with four contigs, a total length of 4,909,184 bp and 100% read mapping coverage (Table 1). MOB-suite version 3.0.3 (10) identified three plasmids: pF12YCO47-2 closely matches *Salmonella enterica* plasmid (HE654725), while pF12YCO47-3 and pF12YCO47-1 closely match *Escherichia coli* plasmids (CP023368 and CP041570). TYGS v391 (11) whole genome comparison assigned F12YCO47 to the species *E. marmotae*. F12YCO47 was classified into clade V by the ClemonTyper webtool version 23.06 (12).

**TABLE 1:** Summary of sequencing, assembly, annotation, antimicrobial resistance, and virulence factor features for *Escherichia marmotae* F12YCO47

Parameters	Data
ONT sequencing	
Number of sequencing reads	115,050
*N*_50_ (bp)	12,840
Mean read length (bp)	10,255.2
Mean contig coverage (x)	159
Mean read quality	20
Assembly	
Number of contigs	4
GC content (%)	50.34
Annotation of chromosome	
Total number of genes	4,798
Protein coding sequences	4,531
RNA (genes)	120
Antibiotic resistance genes	
MdfA	Present
Shiga-toxin genes	Absent
Antimicrobial resistance-associated point mutations	Absent

PGAP identified a total of 4,798 genes in the chromosome, including 4,531 protein coding sequences, 92 tRNAs, 22 rRNAs, 6 ncRNAs, and 2 CRISPR arrays. Genome analysis with ABRIcate version 1.0.0 (https://github.com/tseemann/abricate) identified antimicrobial resistance genes using the ResFinder database (13), chromosomal point mutations using the PointFinder database (14), and virulence genes using the Virulence Factors database (15). ABRIcate identified MdfA as encoding a multidrug efflux pump protein (16).

## Data Availability

The genome sequences are available in GenBank under the accession numbers CP173213, CP173216, CP173214, and CP173215 under BioProject PRJNA1178718 and BioSample SAMN44479625, with raw sequencing data in SRA SRR31403753.
